# Annotations

**Published:** 1890-01-18

**Authors:** 


### Annotations.

**Human Development in Africa.**

The study of the origin and development of man in a remote past is interesting even to fascination. The study of contemporary problems of the same kind ought to be at least as interesting, and certainly is much more practically important. There is no greater mistake than to suppose that the mental evolution of man concerns alone the religionist or the philosopher. Surely the physiologist who seeks to throw light on the functions of the human brain by experiments on living dogs and monkeys cannot afford to despise those functions as displayed in actual life work among the lower races of men. Still less can the practical politician, whose hand may be forced at any time by the contending motions of oppression and resistance among ill-governed peoples, venture to ignore the aspirations of those who are newly awakening to the light of moral responsibility, and the possibilities of civilisation. From every point of view Africa, which has long been the "dark continent," must be looked upon as a vast laboratory, wherein are being worked out some of the most profoundly - interesting problems of advanced human physiology and elementary sociology. It is a fact of striking significance that the introduction of Christianity, a quite recent event, seems to have given an impetus to mental development without parallel in the previous history of Central Africa. The inhabitants of that "vast unknown," who, until within the last fifty years, seem to have been content with what was practically a mere animal existence, have all at once begun to manifest qualities which distinguish the rational and moral human creature. They have received general principles, they have acquired convictions, they have developed a sense of duty and responsibility; and so strong and reliable are these new attainments that their possessors are not only willing to fight for them, but to endure deprivation, cruelty, and death. The letter of Mr. Stanley, published in the newspapers last week, shows that these people have now possessed themselves of a creed which to them is a common rallying point. Each of them, believing in it, is able to trust his fellow believer; and each of them, having acquired what he holds to be a divine charter of rights and liberties, refuses any longer to submit to the irresponsible violence and tyranny of a being lower in the scale of mental evolution than himself. Now these are facts. They are outside the region of dialectic and historical criticism. No German professor in his Weissnichtwo garret can explain them away under a cloud of tobacco smoke. The fact remains, on the authority of the greatest of African explorers, that the native Christians have begun to think, to combine, to destroy tyranny, and to insist upon a government suited to rational and moral creatures. Is this result of high and rapid mental evolution the natural fruit of an understood and honestly accepted Christianity? We believe it is.

**Dr. Underwood's Opinions.**

Dr. Underwood was the American consul in Glasgow, under the late President, and was a very popular man there. Last week he was entertained at a banquet in the corporation galleries by the Pen and Pencil Club; many doctors, lawyers, clergymen, and other citizens being present. In acknowledging the toast of his health, Dr. Underwood expressed the opinion that "in the United States there were greater lawyers at the bar than on the bench, and more learned men in private life than in the nation's councils. It was only accidental when such eminent men as Motley, Lowell, and Phelps represented their country abroad." If he had mentioned doctors he would no doubt have held that there are sometimes abler men and more skilful physicians in country towns and villages than on the medical staffs of metropolitan hospitals. But what then? Is it so common a thing to see the "right man in the right place" in human affairs that we cannot avoid expressions of astonishment when we find it otherwise? The "struggle for life" for the most part consists in the efforts that individuals are more or less consciously making to find their true spheres in the world. The "round man in the square hole" is a sight constantly witnessed. How is this to be remedied? It will never be remedied until there is a general recognition of the fact that the best interests of civilised as well as uncivilised man require in every instance "the tools to be given to him who can handle them." Many clergymen, for example, have no more capacity for preaching or teaching religion than they have for riding horses in Mr. Barnum's circus. Similarly, numbers of doctors would be more congenially employed in ferretting rats or baiting badgers than in the medical treatment of delicate women or nervous children. More primitive peoples are less hampered and injured by this kind of thing than are such comparatively old civilisations as our own. In the youth of nations, when people find themselves in unsuitable callings, they begin afresh at some more congenial occupation. But with us, if a full-grown man gives up a profession with which he is out of sympathy to seek another which better suits his character and disposition, it is considered equivalent to a confession of failure. If there were more manliness among us there would be more elasticity. Men should have the courage of their instincts and convictions, both in private and in public life. They should change their occupations without hesitation if necessity seems to require it, and they should not scruple to remove from all sorts of public positions persons who seem to be injurious to the public interests. These are the "methods of science" applied to common life, and until such methods are universally adopted the coach of the world will always be breaking down and injuring both its drivers and its "fares."

**Gentlemen "Pelicans" and "low organisms."**

The Pelican Club is an object of curious interest at the present moment to students of the human animal in actual life. It seems to consist of members of that particular species of the *genus homo* called "gentlemen." But for scientific purposes the loose and vague use of terms is exceedingly awkward; and it is not improbable that undoubted members of the class "gentlemen," if asked to frame a definition of the term, would elaborate one that would very decisively exclude a large proportion of the members of the Pelican Club. With that, however, we need not concern ourselves. What is interesting to the humanity student is the peculiar connection of this "Gentlemen's" club, as shown by the recent proceedings of its committee, with the noble art of prize-fighting. It has happened that the present writer in the course of his professional avocations has come into personal contact with a good many members of the prize-fighting, cock-fighting, dog-fighting, dog-running, pigeon-flying, and horse-racing fraternities. The experience has been valuable as demonstrating to him the peculiar effects of these various "sports" on the brain and mental and moral functions of the persons engaged in them. Taken as a whole, the "sportsmen" that have to do with "fighting" pastimes belong to a more pronounced type of character than those that have to do with mere "running." The professional running-man, whether his fancy lies with dogs or horses, is more or less of a "modified blackguard," the man who has to do with "fights," in a professional capacity, is a "blackguard outright." The effect produced upon the members of these "brotherhoods" by their peculiar occupations and amusements does not appear to be at all affected by their possession of a liberal education or their want of it. The "educated" associate of the prize ring seems to be quite as much a past master in all the arts of general ruffianism as his uneducated friend and brother. Perhaps it would be a waste of time to deplore and grieve over these peculiar human animals. It is an undoubted fact in the scientific history of man that a certain number of persons seem to be born to ruffianism exactly as others are born to music, to poetry, to mathematics, to mental science, and the like. Good birth, so called, or even high birth, has apparently nothing to do with the matter. There is a retrograde metamorphosis of brain and function which casts a man back in mental development to a stage long prior to Adam—a stage of animal ferocity and unintellectual brutality little, if at all, superior to that of the lower anthropoid apes. It is, perhaps, beyond our province to suggest what should be done to prevent the inevitable mischief which ensues to the race of man from its compulsory association with these "low organisms." In the, possibly remote, future, when national statutes and enactments shall be framed on scientific bases, it will no doubt be found necessary to provide by statute that all the members of these prize-fighting and other similar fraternities shall be knocked on the head, or otherwise disposed of, exactly as we shoot stoats, weasels, and other "vermin."

